# Bowel Bubble Formation with Oral Sulfate Solution for Colonoscopy Bowel Preparation Using Same-Day and Split-Day Regimens: The B-BOSS Study

**DOI:** 10.3390/diagnostics16091391

**Published:** 2026-05-04

**Authors:** Naohisa Yoshida, Ken Inoue, Reo Kobayashi, Yoshikazu Inagaki, Yuri Tomita, Hikaru Hashimoto, Yutaka Inada, Ryohei Hirose, Takeshi Yasuda, Naoto Iwai, Osamu Dohi, Kazuhiko Uchiyama, Hardesh Dhillon, Tomohisa Takagi

**Affiliations:** 1 Department of Molecular Gastroenterology and Hepatology, Graduate School of Medical Science, Kyoto Prefectural University of Medicine, Kyoto 602-8566, Japan; keninoue71@koto.kpu-m.ac.jp (K.I.); reipanna@koto.kpu-m.ac.jp (R.K.); t-yasuda@koto.kpu-m.ac.jp (T.Y.); na-iwai@koto.kpu-m.ac.jp (N.I.); osamu-d@koto.kpu-m.ac.jp (O.D.); k-uchi@koto.kpu-m.ac.jp (K.U.); takatomo@koto.kpu-m.ac.jp (T.T.); 2 Department of Gastroenterology, Nishijin Hospital, Kyoto 602-8319, Japan; y.inaina@s7.dion.ne.jp; 3 Department of Gastroenterology, Kyoto Hakuaikai Hospital, Kyoto 603-8041, Japan; yuri0428@koto.kpu-m.ac.jp; 4 Department of Gastroenterology, Osaka General Hospital of West Japan Railway Company, Osaka 545-0053, Japan; h-hashi@koto.kpu-m.ac.jp; 5 Department of Gastroenterology, Kyoto First Red Cross Hospital, Kyoto 600-8811, Japan; yutak-i@koto.kpu-m.ac.jp; 6 Department of Infectious Disease, Graduate School of Medical Science, Kyoto Prefectural University of Medicine, Kyoto 602-8566, Japan; ryo-hiro@koto.kpu-m.ac.jp; 7 Department of Gastroenterology, Barwon Health, Victoria 3220, Australia; hardeshdhillon92@gmail.com

**Keywords:** bowel preparation, colonoscopy, oral sulfate solution, bowel bubble

## Abstract

**Background and Objectives:** Bowel bubble formation during colonoscopy can impair mucosal visualization and reduce procedural efficiency. However, its clinical significance remains incompletely characterized. This study aimed to evaluate the prevalence of severe bowel bubbles in the proximal colon and their impact on colonoscopic quality in patients undergoing bowel preparation with oral sulfate solution (OSS), using either a same-day regimen (SAR) with 480 mL OSS or a split-day regimen (SPR) with 960 mL OSS. **Methods:** This retrospective study was conducted between October 2024 and December 2025 across two affiliated institutions. Patients who underwent colonoscopy with OSS-based bowel preparation were included. SAR was used for screening, symptomatic evaluation, and surveillance colonoscopy. SPR was applied exclusively to patients with a prior history of sodium picosulfate-related abdominal pain or inadequate bowel preparation with SAR. Sodium picosulfate was prescribed on the day before colonoscopy in the SAR group and a low-residual diet was administered in both the SAR and SPR groups. The prevalence of severe bubbles in the proximal colon was evaluated, and their impact on colonoscopic quality was examined. **Results:** A total of 176 SAR cases and 51 SPR cases were analyzed. The rate of severe bubbles in the proximal colon was identical between both regimens (17.6%). Compared with cases without severe bubbles, those with severe bubbles had significantly longer cecal insertion times (median [IQR]: 7.0 [5.0–10.0] vs. 9.0 [7.0–13.0] min, *p* = 0.018) and total procedure time (20.0 [16.0–25.0] vs. 24.0 [19.0–30.0] min, *p* = 0.024). Preparation-to-colonoscopy time was also longer in cases with severe bubbles (5.0 [4.0–5.5] vs. 5.0 [5.0–6.0] h, *p* = 0.041). Adenoma detection rates were 73.2% in cases without severe bubbles and 67.5% in those with severe bubbles (*p* = 0.571). **Conclusions:** Severe bowel bubble formation tended to be associated with longer cecal insertion and total procedure times, and was more frequently observed with a longer preparation-to-colonoscopy interval.

## 1. Introduction

Adequate bowel preparation is a critical determinant of colonoscopy quality [1,2]. Among available bowel preparation agents, polyethylene glycol (PEG) has been widely used for decades owing to its effective cleansing efficacy and favorable safety profile. A key limitation of PEG-based preparation, however, is the requirement for ingestion of a large solution volume, typically up to 4 L. Two principal regimens employed for bowel preparation include a same-day regimen (SAR) and a split-day regimen (SPR). Current guidelines preferentially recommend SPR on the basis of superior bowel cleansing and better patient tolerance [1,2].

Oral sulfate solution (OSS) was developed as a bowel preparation agent in 2010. In Japan, it is supplied as two 480 mL bottles. Following ingestion of each bottle, patients are instructed to consume approximately 1 L of additional water, resulting in a total fluid intake of up to approximately 3 L. A Japanese randomized controlled trial (RCT) demonstrated that bowel cleansing quality with SAR was non-inferior to SPR using OSS [3]. In addition, a meta-analysis showed that OSS achieved superior bowel preparation quality compared with PEG-based solutions [4]. Our group previously reported the effectiveness and tolerability of a novel SAR protocol employing 480 mL OSS in combination with a liquid diet and sodium picosulfate on the day prior to colonoscopy [5].

Bowel bubble formation represents an under-recognized but clinically relevant factor influencing overall colonoscopy quality. Simethicone supplementation of a bowel preparation solution has been shown in several studies to reduce the bubble burden though this use is not formally approved in Japan [6,7]. We previously reported that bowel bubbles may occur more frequently with OSS than with high-volume PEG (H-PEG) [8]; however, that study was exploratory in nature and was limited by a small OSS cohort. The true prevalence of bowel bubble formation with OSS-based bowel preparation remains to be defined. The present study aimed to evaluate bowel bubble formation associated with OSS for colonoscopy and its association with colonoscopy quality indicators in both SAR and SPR: The B-BOSS study.

## 2. Methods

### 2.1. Study Design and Patients

**Figure 1:**
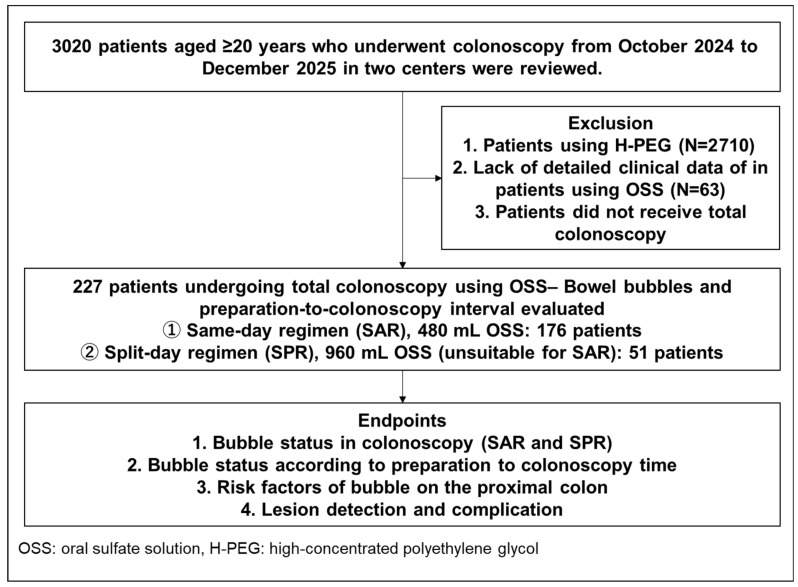
A flow diagram of the present study.

This retrospective cohort study was conducted between October 2024 and December 2025 at two Japanese institutions. We reviewed the medical records of 3,020 patients aged ≥20 years who underwent colonoscopy during this period (Figure 1). Patients who underwent OSS-based bowel preparation using either SAR or SPR, and in whom bowel bubble status was formally evaluated during colonoscopy, were eligible for inclusion.

The detailed inclusion criteria of this study were as follows: (1) colonoscopy performed for screening, surveillance, or symptomatic evaluation; and (2) patients with an estimated glomerular filtration rate (eGFR) ≥ 30 mL/min who agreed to OSS use for SAR and SPR bowel preparation. SPR was administered exclusively to patients with a prior history of sodium picosulphate-related abdominal pain or inadequate bowel preparation with a same-day preparation using 1 L H-PEG or 480 mL OSS, based on our previously published protocols [5,8]. Cases in which cold snare polypectomy was performed for benign lesions less than 10 mm in size were also included [9].

Exclusion criteria were as follows: (1) The use of H-PEG for bowel preparation; (2) incomplete clinical data, (3) An incomplete colonoscopy and 4. procedures involving endoscopic mucosal resection and endoscopic submucosal dissection.

### 2.2. Outcomes

The primary outcome of this study was the presence and severity of bowel bubbles during colonoscopy. Bowel bubble status was evaluated in the proximal colon, distal colon, and rectum using the Bubble Scoring System for Colonoscopy (BBS-C), according to our previous report [8].

Secondary outcomes included bowel preparation quality, cleansing time, preparation-to-colonoscopy time, polyp detection, and severe adverse events. In the SPR group, the time from OSS intake on the evening prior to colonoscopy to the completion of bowel evacuation was also analyzed. Clinical and procedural characteristics including risk factors related to severe bubbles in the proximal colon were examined. Given that the inclusion criteria differed substantially between the SAR and SDR groups, no direct statistical comparison between regimens was intended. Rather, the aim of this study was to characterize the real-world prevalence of bowel bubbles and associated colonoscopic results in each regimen independently. Accordingly, no adjustment for confounding factors was performed.

### 2.3. Bubble Scoring System of Colonoscopy, BBS-C

Several scoring systems for grading bowel bubbles during colonoscopy have been previously described [10,11,12]. The Bubble Scoring System for Colonoscopy (BBS-C), was developed by our group as a more stringent classification tool, with a particular emphasis on the grading of severe bubble burden [8].

**Figure 2:**
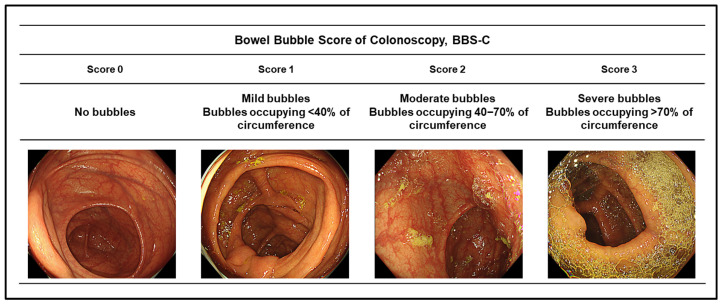
Bowel bubble scoring of colonoscopy: BSS-C.

Bowel bubble assessment was performed according to colonic segments (proximal colon and distal colon) without prior administration of simethicone. The BBS-C grades bubble burden according to the proportion of luminal circumference occupied by bubbles as follow: score 0: no bubbles, score 1: bubbles occupying <40% of the luminal circumference, score 2: bubbles occupying 40–70% of the luminal circumference, score 3: bubbles occupying >70% of the luminal circumference (severe bubbles) (Figure 2). The worst score observed within each segment was recorded. Although the existing scale defines severe bubbles as involvement of ≥50% of the visual field, this threshold may include cases that do not interfere with the examination [10]. Therefore, in our BBS-C, we applied a stricter definition, setting severe bubbles as the involvement of >70% of the visual field to capture only those that could potentially impair the procedure. Our previous study showed that the inter-observer agreement rate of the BBS-C among three physicians was 75% (75/100) in an internal validation [8]. In the present study, BBS-C scores were independently assessed by three physicians (N.Y., Y.I. and R.K.), with discrepant cases resolved by consensus review.

### 2.4. Colonoscopic Evaluation

Patient data were retrieved from an electronic medical record database. Baseline information included age, sex, medication use (antispasmodics and antithrombotics), and a history of colorectal surgery. Colonoscopic variables included sedation use, insertion time, total procedure time, bowel preparation quality, severe pain, diverticula, the adenoma detection rate (ADR), and the sessile serrated lesion (SSL) detection rate.

Bowel preparation quality was assessed using the Aronchick Bowel Preparation Scale in accordance with the Japan Gastroenterological Endoscopy Society guidelines [13]. The Aronchick scale is a validated five-point grading system: 1 = excellent, 2 = good, 3 = fair, 4 = poor, and 5 = inadequate [14]. Scores of 1 or 2 were classified as good bowel preparation, whereas scores of 3 or 4 were defined as poor bowel preparation. Acceptable bowel preparation was defined as scores of 1–3.

Image-enhanced endoscopy, including blue laser/light imaging (BLI), linked color imaging (LCI), or narrow-band imaging (NBI) was employed alongside white-light imaging (WLI) for polyp detection and characterization, at the discretion of the performing endoscopist [15,16].

The proximal colon was defined as the cecum, ascending colon, and transverse colon. The distal colon was defined as the descending colon, sigmoid colon, and rectum. All colonoscopies were performed by four expert endoscopists, defined as those with experience of more than 1000 colonoscopies, including at least 50 withdrawal examinations using LCI or NBI [16]. Total procedure time encompassed cecal insertion time, withdrawal time, and polypectomy time where applicable.

Regarding adverse events, severe periprocedural and delayed bleeding and perforation were recorded. Delayed bleeding was defined as post-procedural bleeding requiring endoscopic hemostasis or a decrease in hemoglobin of more than 2 g/dL within 30 days after the procedure [17]. Delayed perforation was defined as the presence of free air detected by computed tomography within 14 days of the procedure, in the absence of an intraprocedural perforation [17].

The histopathological diagnosis of lesions, including adenomas and SSLs, was established according to the 2019 WHO classification [18].

### 2.5. Endoscopic Equipment

Colonoscopes used in this study included the EC-L600ZP7, EC-760ZP, and EC-860ZP (Fujifilm Co., Tokyo, Japan), operated with the EP-8000 or VP-7000 processor system (Fujifilm). Additional colonoscopes included the PCF-H290AZI, CF-XZ1200L/I, and CF-EZ1500DL/I (Olympus Co., Tokyo, Japan) operated with the CV-1500 or CV-290 system (Olympus). A water-jet system and CO_2_ insufflation (GW-100; Fujifilm, Tokyo, Japan) were employed in all procedures. A diluted simethicone solution was administered through the water-jet system or the endoscopic channel as clinically indicated during the procedure.

### 2.6. Bowel Preparation

For SAR, patients followed a low-residue diet on the day before colonoscopy and took 10 mL sodium picosulfate at 9 p.m. On the morning of colonoscopy, 480 mL of OSS (SULPREP; Fuji Pharma Co., Ltd., Tokyo, Japan) was ingested three to four hours prior to the procedure, together with 960 mL of water, with the entire intake completed within 90 min.

If adequate bowel preparation was not achieved within 4–5 h, one or two enemas were administered. Cleansing time was defined as the interval from the commencement of OSS ingestion on the day of colonoscopy to the point when rectal effluent became clear.

For SPR, 480 mL of OSS was administered at 7 p.m. on the evening before colonoscopy in place of sodium picosulfate. On the day of colonoscopy, another 480 mL of OSS was administered similarly to SAR. The two-step OSS and water ingestion method was identical to that used in SAR. Preparation-to-colonoscopy time was defined as the interval from the commencement of OSS ingestion on the day of colonoscopy to the start of colonoscopy.

### 2.7. Ethical Statement

This study was conducted as a retrospective subgroup analysis within a multicenter prospective and retrospective study organized by our department. The study protocol was approved by the Ethics Committee of Kyoto Prefectural University of Medicine (ERB-C-1704-3, approval date: 29 June 2021) as part of a large-scale prospective and retrospective endoscopic study and was conducted in accordance with the Declaration of Helsinki.

Informed consent was obtained using an opt-out method through notifications posted on the hospital website and within the endoscopy unit.

### 2.8. Statistical Analysis

Categorical variables were expressed as numbers and percentages. Continuous variables such as age, which followed a normal distribution were expressed as mean ± standard deviation (SD), while cecal insertion time, total procedure time, cleansing time, and preparation-to-colonoscopy time were expressed as median [interquartile range (IQR)].

The Mann–Whitney U test was used to compare continuous variables between two independent groups, and the chi-square test was used to compare categorical variables. Multivariate logistic regression analysis was performed to identify risk factors for severe proximal colonic bubbles. Variables with a *p*-value < 0.20 in the univariate analysis were included in the multivariate analysis.

All statistical analyses were performed using SPSS software (version 22.0 for Windows; IBM Japan Ltd., Tokyo, Japan). A *p*-value < 0.05 was considered statistically significant.

## 3. Results

Among the 3,020 patients reviewed, 176 SAR cases and 51 SPR cases met the inclusion criteria and were analyzed (Figure 1, Table 1). In the SPR group, the indications for SPR use were inadequate bowel preparation on a prior colonoscopy in 33 patients and sodium picosulfate-related abdominal pain during previous bowel preparation in 18 patients. In the SAR and SPR groups, mean age (±SD) was 68.3 ± 12.4 and 67.7 ± 11.9 years and the proportions of male patients were 43.2% and 58.8%, respectively. Rates of acceptable bowel preparation were 97.2% and 98.0% in the SAR and SPR groups, respectively. No periprocedural and post-procedural severe adverse events were recorded in either group.

Cleansing time (median [IQR]) was 2.0 [1.8–3.0] h in the SAR group and 2.7 [2.5–4.0] h in the SPR group (Table 2). The preparation-to-colonoscopy time was 5.0 [4.0–5.5] h and 5.0 [4.5–6.0] h in the SAR and SPR groups, respectively. In the SPR group, the cleansing time on the day prior to colonoscopy was 3.0 [3.0–4.0] h. The prevalence of severe bubbles in the proximal colon (BBS-C score 3) were 17.6% in both the SAR and SPR groups.

When stratified by preparation-to-colonoscopy time, the rates of severe bubbles (BBS-C score 3) in the proximal colon were 10.3% and 20.1% for ≤4 h and >4 h, respectively (*p* = 0.091) (Table 3). In the SAR group, the rates were 8.9% and 19.8% (*p* = 0.074), and in the SPR group, the rates were 15.4% and 18.4%, respectively (*p* = 0.862).

Clinical and procedural characteristics, including risk factors related with severe bubbles in the proximal colon were examined using logistic regression analysis (Table 4). On univariate analysis, preparation-to-colonoscopy time (median [IQR]) was significantly longer in patients with severe bubbles compared with those without (5.0 [5.0–6.0] vs. 5.0 [4.0–5.5] h, *p* = 0.041). Multivariate logistic regression analysis demonstrated that preparation-to-colonoscopy time was an independent risk factor for severe bubbles in the proximal colon (adjusted odds ratio [OR], 1.38; 95% confidence interval [CI], 1.04–1.85; *p* = 0.028).

The ADR and SSL detection rates stratified by BBS-C score were also examined (Table 5). For BBS-C scores 0 and 3, ADRs were 73.2% and 67.5% (*p* = 0.571), and SSL detection rates were 13.4% and 15.0% (*p* = 0.812), respectively.

Adverse events occurred in 8.8% of all patients, 9.1% in the SAR group, and 7.8% in the SPR group (Table 6). No adverse events necessitated pharmacological intervention or hospital admission. Two cases of abdominal pain were attributed to sodium picosulfate administered the day prior to colonoscopy. Notably, among the 18 patients with a history of abdominal pain due to sodium picosulfate, no abdominal pain occurred with OSS intake. The rate of sleep disturbance was significantly higher in the SPR group than in the SAR group (19.8% vs. 3.4%, *p* < 0.001).

## 4. Discussion

The prevalence of severe bowel bubbles (BBC-S Score 3) in the present study was 17.6% in both the SAR (176 cases) and SPR (51 cases) using OSS. This rate is higher than those reported in previous studies. A study from China reported severe bubbles and no bubbles in 8.9% and 34.3% of 4940 cases (mean ± SD: 61.4 ± 10.3 years), respectively, using a published subjective scoring system ranging from 0 (none) to 3 (severe) [12,19]. All patients in that study received a split-day regimen (SPR) of 3 L of polyethylene glycol (PEG). A further study of 304 patients (mean ± SD: 48.74 ± 11.73 years) using 3 L of PEG reported a severe bubble rate of 11.6% based on the same scoring system [12,20].

In our previous study using the BBS-C, severe bubbles occurred in 11.1% of 45 OSS cases (480 mL regimen) with SAR and in 17.9% of 117 H-PEG cases (1 L regimen) (After matching: 11.1% vs. 6.7%) [8]. However, direct comparison across studies is limited by heterogeneity in patient demographics, bowel preparation regimens and solutions, rates of adequate bowel preparation, and the scoring systems employed. Thus, further studies using a standardized methodology are needed to establish the true prevalence of bowel bubbles.

Only a limited number of studies have examined risk factors for severe bowel bubbles. One study reported that bubbles were more frequently observed with 1 L PEG than with 2 L PEG [21]. Another identified age 45–60 years (OR 2.09, 95% CI: 1.13–3.87), age >60 years (OR 1.99, 95% CI: 1.01–3.95), and anxiety (OR 3.85, 95% CI: 2.12–6.97) as independent risk factors [20]. In our previous study, antithrombotic drug use was significantly associated with a lower prevalence of severe bubbles in the proximal colon on univariate analysis (*p* = 0.043) [8], though another study found no significant association between antithrombotic use and bubble scores [22].

Our previous study also noted an association between good bowel preparation and severe bubbles [8]. We observed that cases with severe bubbles in the small intestine frequently demonstrated a concurrent bubble burden in the proximal colon, suggesting that bubbles may originate from the small intestine, driven by strong peristalsis induced by the cleansing solution—the same mechanism responsible for fecal excretion. Residual bubbles without fecal residue might consequently be interpreted as good bowel preparation. A prolonged preparation-to-colonoscopy interval may further contribute to severe bubble accumulation, which was a significant independent risk factor in the present study. The mechanisms underlying bowel bubble formation warrant further investigation.

Previous studies have reported conflicting findings regarding the impact of bubble burden on the ADR. While some studies found no effect of bubble scores on ADR [22,23], others demonstrated an association between severe bubble scores and a lower ADR and advanced ADR [18,24]. Consistent with our previous study, the present study found no significant association between the BBC-S and ADR or the SSL detection rate [8].

Regarding other clinical impacts of bowel bubbles, an RCT comparing a placebo with simethicone supplementation demonstrated reduced bubble burden in the simethicone group but no difference in total procedure time (28.7 vs. 28.5 min) [22]. In contrast, the present study found that severe bubbles were associated with longer insertion and total procedure times, suggesting a measurable impact on procedural efficiency. Further investigations are required to clarify the clinical significance of bowel bubbles during colonoscopy.

With respect to OSS-related adverse events, a Japanese RCT reported adverse event rates of 4.0% (8/200) in the SAR group and 9.4% (19/202) in the SPR group, comprising mild gastrointestinal symptoms, including nausea, vomiting, and nasopharyngitis [3]. A systematic review of seven RCTs comparing OSS with H-PEG showed that OSS was associated with a 35% increased risk of nausea (RR 1.35, 95% CI 1.03–1.77, *p* = 0.03) and more than double the risk of vomiting (RR 2.30, 95% CI 1.63–3.23, *p* < 0.05) [4]. Our previous study documented mild dehydration following OSS intake, with hematocrit values increasing from 42.3 ± 3.8 before to 43.2 ± 4.0 after 480 mL of OSS administration, while serum electrolytes, including magnesium remained stable. Another study comparing 1 L H-PEG and OSS also demonstrated significant increases in serum creatinine levels in both groups (1 L H-PEG: 0.77 vs. 0.91, *p* < 0.001; OSS: 0.78 vs. 0.81, *p* = 0.04) [25]. In the current study, the overall rate of adverse events was 8.8%, with no significant difference between the SAR and SPR groups (9.1% vs. 7.8%). Notably, among 18 patients who had previously experienced sodium picosulfate-related abdominal pain, no abdominal pain was reported following OSS ingestion in the SPR protocol. These findings suggest that SPR with OSS may represent a well-tolerated alternative bowel preparation strategy in this patient subgroup.

The cleansing time for SAR with OSS in a Japanese RCT was 170.2 ± 57.4 min, which was longer than the median cleansing time observed in the present study (2.0 [1.8–3.0] h) [3]. This discrepancy may be attributed to the reduced OSS volume used in our SAR. The relatively short cleansing time observed in the present study has practical implications; patients scheduled for morning colonoscopy may not require early waking, potentially reducing sleep disruption compared with conventional SPR. This protocol may also be particularly advantageous for urgent colonoscopy, where rapid bowel preparation is desired.

Several limitations of this study warrant acknowledgement. First, this was a retrospective study with a relatively small sample size, limiting statistical power. Second, patient enrollment was non-consecutive and bowel preparation regimens were selected by individual physicians, introducing a potential risk of selection bias. Third, the 480 mL OSS regimen was evaluated only in a Japanese population, whose body habitus is generally smaller than that of Western populations, which may limit the generalizability of the findings. Fourth, the primary purpose of our study was not a direct comparison between the SAR and SPR groups; accordingly, no statistical adjustment for confounding variables was performed and multivariate logistic regression was not conducted for between-group comparisons. Fifth, bowel preparation quality was not assessed in each by colonic segments, as the Aronchick Bowel Preparation Scale yields a global rather than a segmental score. Sixth, long-term outcomes, including the recurrence of the lesions resected via polypectomy, were not examined. Seventh, withdrawal time was not assessed in this study, although it has been reported to be significantly associated with the ADR [26]. Finally, formal external validation of the BBS-C was not performed in the current study and therefore the prevalence rates of bubbles and other analyses reported in this study should be interpreted with this limitation in mind. Prospective validation in larger, independent cohorts is warranted. Therefore, the ADRs reported here should be interpreted with this limitation in mind.

## 5. Conclusions

Severe bowel bubble formation tended to be associated with longer cecal insertion and total procedure times, and was more frequently observed with a longer preparation-to-colonoscopy interval. Optimizing colonoscopy scheduling relative to the completion of bowel preparation may help reduce the severe bubble burden and improve procedural efficiency.

## Figures and Tables

**Table 1:** Patients’ characteristics in the SAR and SPR group.

Patient Characteristics	SAR 480 mL of OSS	SPR 960 mL of OSS
Case number	176	51
Age, mean ± SD	68.3 ± 12.4	67.7 ± 11.9
Age, *n* (%)<80/≥80	28 (62.2)/17 (37.8)	68 (58.1)/49 (41.9)
Sex, *n* (%) Male/Female	76 (43.2)/100 (56.8)	30 (58.8)/21 (41.2)
Antispasmodics, *n* (%)	144 (81.8)	44 (86.3)
Antithrombotics, *n* (%)	27 (15.3)	6 (11.8)
Colorectal surgery	12 (15.0)	8 (10.0)
Sedation, *n* (%)	67 (38.1)	27 (52.9)
Insertion time, min, median [IQR]	8.5 [6.0–12.0]	7.0 [5.0–10.0]
Total procedure time, min, median [IQR]	23.0 [18.0–28.0]	20.0 [16.0–25.0]
Excellent bowel preparation (Aronchick score 1), *n* (%)	45 (25.6)	17 (33.3)
Good bowel preparation (Aronchick score: 1 or 2), *n* (%)	144 (81.8)	43 (84.3)
Acceptable bowel preparation (Aronchick score 1 to 3), *n* (%)	172 (97.2)	50 (98.0)
Severe pain, *n* (%)	4 (2.3)	1 (2.0)
Diverticula, *n* (%)	25 (31.3)	18 (22.5)
ADR *n* (%) SSL detection rate, *n* (%)	121 (68.8) 24 (13.6)	36 (70.6) 5 (9.8)

SAR: same-day regimen, SPR: sprit-day regimen, OSS: oral sulfate solution, IQR: interquartile, proximal colon: cecum to transvers colon, ADR: adenoma detection rate, SSL: sessile serrated lesions.

**Table 2:** The cleansing time and BBS-C in the SAR and SPR groups.

	SAR 480 mL OSS	SPR 960 mL OSS
Cleansing time, hour, median [IQR] (Mean ± SD)	2.0 [1.8–3.0] 2.3 ± 0.7	2.7 [2.5–4.0] 2.6 ± 0.7
Preparation to colonoscopy time, hour, median [IQR] (mean ± SD)	5.0 [4.0–5.5] (5.0 ± 1.1)	5.0 [4.5–6.0] (5.1 ± 1.4)
Cleansing time one day prior to colonoscopy, hour, median [IQR] (mean ± SD)	N/A	3.0 [3.0–4.0] (3.8 ± 1.8)
Proximal colon,		
BBS-C score, *n* (%)		
Score 0	62 (35.2)	20 (39.2)
Score 1	49 (27.8)	13 (25.5)
Score 2	34 (19.3)	9 (17.6)
Score 3	31 (17.6)	9 (17.6)
Distal colon,		
BBS-C score, *n* (%)		
Score 0	122 (69.3)	31 (60.8)
Score 1	34 (19.3)	16 (31.4)
Score 2	9 (5.1)	1 (2.0)
Score 3	11 (6.3)	3 (5.9)

BBS-C: Bowel Bubble Score of Colonoscopy, IQR: interquartile, proximal colon: SD: standard deviation, N/A: not applicable, cecum to transvers colon, distal colon: descending colon to rectum.

**Table 3:** The comparison of colonoscopic status between ≤4 h and >4 h of preparation to colonoscopy.

	Preparation-to- Colonoscopy Time ≤ 4 h	Preparation-to- Colonoscopy Time > 4 h	*p*-Value
Case number, overall	58	169	
Proximal colon,			0.091
BBS-C, *n* (%)			
Score 0	17 (29.3)	65 (38.5)	
Score 1	18 (31.0)	44 (26.0)	
Score 2	17 (29.3)	26 (15.4)	
Score 3	6 (10.3)	34 (20.1)	
Case number, SAR	45	131	
Proximal colon,			0.074
BBS-C, *n* (%)			
Score 0	15 (33.3)	47 (35.9)	
Score 1	14 (31.1)	35 (26.7)	
Score 2	12 (26.7)	22 (17.6)	
Score 3	4 (8.9)	27 (19.8)	
Case number, SPR	13	38	
Proximal colon,			0.862
BBS-C, *n* (%)			
Score 0	2 (15.4)	18 (47.4)	
Score 1	4 (30.7)	9 (23.7)	
Score 2	5 (38.5)	4 (10.5)	
Score 3	2 (15.4)	7 (18.4)	

BBS-C: Bowel Bubble Score of Colonoscopy, proximal colon: cecum to transvers colon, SAR: same-day regimen, SPR; sprit-day regimen.

**Table 4:** Clinical and procedural characteristics including risk factors related with severe bubbles in the proximal colon.

	Univariate Analysis			Multivariate Analysis		
	No Severe Bubble (*n* = 187)	Severe Bubble (*n* = 40)	*p*-Value	Adjusted OR	95% CI	*p*-Value
Age, mean ± SD	68.0 ± 12.4	69.2 ± 11.9	0.579	1.01	0.98–1.04	0.642
Sex (female), *n* (%)	91 (48.7)	16 (40.0)	0.384	0.72	0.34–1.51	0.379
Antispasmodics, *n* (%)	156 (83.4)	32 (80.0)	0.645	N/A		
Antithrombotics, *n* (%)	29 (15.5)	4 (10.0)	0.465	N/A		
Colorectal surgery, *n* (%)	22 (11.8)	3 (7.5)	0.583	N/A		
Diverticula, *n* (%)	81 (43.3)	15 (37.5)	0.598	N/A		
Sedation, *n* (%)	82 (43.9)	12 (30.0)	0.115	0.66	0.30–1.44	0.295
Insertion time, min, median [IQR]	7.0 [5.0–10.0]	9.0 [7.0–13.0]	0.018	N/C		
Total procedure time, min, median [IQR]	20.0 [16.0–25.0]	24.0 [19.0–30.0]	0.024	N/C		
Bad preparation, *n* (%)	35 (18.7)	5 (12.5)	0.493	N/A		
Severe pain, *n* (%)	5 (2.7)	0 (0.0)	0.589	N/A		
Group, SAR/SPR, *n* (%)	145/42 (77.5/22.5)	31/9 (77.5/22.5)	1.000	N/A		
Preparation-to-colonoscopy time (h), median [IQR] (mean ± SD)	5.0 [4.0–5.5] 4.9 ± 1.1	5.0 [5.0–6.0] 5.4 ± 1.3	0.041	1.38	1.04–1.85	0.028

SD: standard deviation, IQR: interquartile, SAR: same-day regimen, SPR; sprit-day regimen.

**Table 5:** The comparison of lesion detection according to BBS-C.

	BBS-C Score 0 *N* = 82	BBS-C Score 1 *N* = 62	BBS-C Score 2 *N* = 43	BBS-C Score 3 *N* = 40	*p*-Value Score 0 vs. Score 3
ADR, *n* (%)	60 (73.2)	40 (64.5)	30 (69.8)	27 (67.5)	0.571
SSL detection, *n* (%)	11 (13.4)	6 (9.7)	6 (14.0)	6 (15.0)	0.812

BBS-C: Bowel Bubble Score of Colonoscopy, ADR: adenoma detection rate, SSL: sessile serrated lesions.

**Table 6:** Adverse events in the SAR and SPR group.

	Case Number	Adverse Events	Nausea	Vomiting	Abdominal Discomfort	Abdominal Pain	Sleeplessness
Overall cases	227	20 (8.8)	8 (3.5)	8 (3.5)	2 (0.9)	2 (0.9)	16 (7.0)
SAR	176	16 (9.1)	6 (3.4)	7 (4.0)	1 (0.6)	2 (1.1)	6 (3.4)
SPR	51	4 (7.8)	2 (3.9)	1 (2.0)	1 (2.0)	0 (0.0)	10 (19.8)
Sub-analysis SPR for previous abdominal pain	18	1 (5.6)	1 (5.6)	0 (0.0)	0 (0.0)	0 (0.0)	4 (22.2)
Sub-analysis SPR for poor preparation	33	3 (9.1)	1 (3.0)	1 (3.0)	1 (3.0)	0 (0.0)	6 (18.2)

SAR: same-day regimen, SPR: sprit-day regimen.

## Data Availability

The patient data used to support the findings of this study are available from the corresponding author upon request. However, some data is restricted by the institutional review board of the Kyoto Prefectural University of Medicine.

## Abbreviations

ADR	adenoma detection rate
BBS-C	Bubble Scoring System for Colonoscopy
BLI	blue laser/light imaging
CI	confidence interval
CO_2_	carbon dioxide
eGFR	estimated glomerular filtration rate
H-PEG	high-volume polyethylene glycol
IQR	interquartile range
LCI	linked color imaging
N/A	not applicable
NBI	narrow-band imaging
OR	odds ratio
OSS	oral sulfate solution
PEG	polyethylene glycol
RCT	randomized controlled trial
SAR	same-day regimen
SD	standard deviation
SPR	split-day regimen
SSL	sessile serrated lesion
WLI	white-light imaging
